# Cross-Border Transmission of *Salmonella* Choleraesuis var. Kunzendorf in European Pigs and Wild Boar: Infection, Genetics, and Evolution

**DOI:** 10.3389/fmicb.2019.00179

**Published:** 2019-02-06

**Authors:** Pimlapas Leekitcharoenphon, Gitte Sørensen, Charlotta Löfström, Antonio Battisti, Istvan Szabo, Dariusz Wasyl, Rosemarie Slowey, Shaohua Zhao, Anne Brisabois, Christian Kornschober, Age Kärssin, Jánosi Szilárd, Tomáš Černý, Christina Aaby Svendsen, Karl Pedersen, Frank M. Aarestrup, Rene S. Hendriksen

**Affiliations:** ^1^European Union Reference Laboratory for Antimicrobial Resistance, WHO Collaborating Center for Antimicrobial Resistance in Food Borne Pathogens and Genomics, Research Group for Genomic Epidemiology, National Food Institute, Kongens Lyngby, Denmark; ^2^RISE Research Institutes of Sweden, Lund, Sweden; ^3^Istituto Zooprofilattico Sperimentale del Lazio e della Toscana, National Reference Laboratory for Antimicrobial Resistance, Rome, Italy; ^4^National Salmonella Reference Laboratory, Unit Molecular Microbiology and Genome Analysis, Federal Institute for Risk Assessment, Berlin, Germany; ^5^National Veterinary Research Institute Department of Microbiology, National Reference Laboratory for Salmonellosis and Antimicrobial Resistance, Puławy, Poland; ^6^Department of Agriculture, Food and the Marine Laboratories, Celbridge, Ireland; ^7^Division of Animal and Food Microbiology, Center for Veterinary Medicine, United States Food and Drug Administration, Laurel, MD, United States; ^8^French Agency for Food, Environmental and Occupational Health and Safety, Maisons-Alfort, France; ^9^NRC Salmonella, Austrian Agency for Health and Food Safety, Graz, Austria; ^10^Veterinaar- ja Toidulaboratoorium, Tartu, Estonia; ^11^Bakteriológiai Laboratórium, Állategészségügyi Diagnosztikai Igazgatóság, Nemzeti Élelmiszerlánc-biztonsági Hivatal, Budapest, Hungary; ^12^Státní Veterinární Ústav Praha, Prague, Czechia; ^13^National Veterinary Institute, Technical University of Denmark, Kongens Lyngby, Denmark

**Keywords:** *Salmonella* Choleraesuis, Kunzendorf, epidemiology, whole genome sequencing, phylogenetics, transmission, wild boar, antimicrobial resistance genes

## Abstract

*Salmonella enterica* subspecies *enterica* serotype Choleraesuis is a swine adapted serovar. *S.* Choleraesuis variant Kunzendorf is responsible for the majority of outbreaks among pigs. *S.* Choleraesuis is rare in Europe, although there have been serious outbreaks in pigs including two outbreaks in Denmark in 1999–2000 and 2012–2013. Here, we elucidate the epidemiology, possible transmission routes and sources, and clonality of European *S*. Choleraesuis isolates including the Danish outbreak isolates. A total of 102 *S*. Choleraesuis isolates from different European countries and the United States, covering available isolates from the last two decades were selected for whole genome sequencing. We applied a temporally structured sequence analysis within a Bayesian framework to reconstruct a temporal and spatial phylogenetic tree. MLST type, resistance genes, plasmid replicons, and accessory genes were identified using bioinformatics tools. Fifty-eight isolates including 11 out of 12 strains from wild boars were pan-susceptible. The remaining isolates carried multiple resistance genes. Eleven different plasmid replicons in eight plasmids were determined among the isolates. Accessory genes were associated to the identified resistance genes and plasmids. The European *S*. Choleraesuis was estimated to have emerged in ∼1837 (95% credible interval, 1733–1983) with the mutation rate of 1.02 SNPs/genome/year. The isolates were clustered according to countries and neighbor countries. There were transmission events between strains from the United States and European countries. Wild boar and pig isolates were genetically linked suggesting cross-border transmission and transmission due to a wildlife reservoir. The phylogenetic tree shows that multiple introductions were responsible for the outbreak of 2012–2013 in Denmark, and suggests that poorly disinfected vehicles crossing the border into Denmark were potentially the source of the outbreak. Low levels of single nucleotide polymorphisms (SNPs) differences (0–4 SNPs) can be observed between clonal strains isolated from different organs of the same animal. Proper disinfection of livestock vehicles and improved quality control of livestock feed could help to prevent future spread of *S*. Choleraesuis or other more serious infectious diseases such as African swine fever (ASF) in the European pig production system.

## Introduction

*Salmonella enterica* is a common cause of human gastroenteritis and bacteremia worldwide (Helms et al., 2005), and many animals species, particularly food animals, have been identified as reservoirs for non-typhoid *Salmonella* (Coyle et al., 1988; Humphrey and Hart, 1988). Although approximately 2,600 serovars of *Salmonella enterica* have been identified, most human infections are caused by a limited number of serovars (Hendriksen et al., 2011).

*Salmonella enterica* subspecies *enterica* serotype Choleraesuis is a serovar adapted to pigs but it also has a propensity to cause extraintestinal infections in humans (Chiu et al., 2004; Sirichote et al., 2010). Pigs are normally asymptomatic carriers, but *S.* Choleraesuis may also cause clinical salmonellosis and paratyphoid (Griffith et al., 2006). Based on H_2_S production and mucate utilization, *S.* Choleraesuis has been classified into variants sensu stricto, Decatur and Kunzendorf, of which *S.* Choleraesuis var. Kunzendorf is responsible for the majority of outbreaks among pigs (Fedorka-Cray et al., 2000).

It has been generally accepted that *S*. Choleraesuis originated from the Western Hemisphere (Barnes and Sorensen, 1975). *S.* Choleraesuis was first isolated and described by Salmon and Smith in 1886 in the United States from cases of “hog cholera” and, since they experimentally produced with this bacterium a disease condition which resembled hog cholera, for many years it was considered to be the cause of the disease. Years later, Schweinitz and Dorset (1903), and Dorset (1905) conclusively demonstrated a filterable virus as the cause of hog cholera (Biester, 1958). By 1940, Bruner and Edwards reported *S*. Choleraesuis var. Kunzendorf as the predominant type of *Salmonella* spp. isolated from swine in the United States (Biester, 1958). Among more recent reports in Eastern Asia, a fluoroquinolone resistant strain emerged around 1998–2004 in humans and pigs in Taiwan (Chiu et al., 2002). In Europe, *S.* Choleraesuis is not considered a dominant serovar, in slaughter pigs and breeding herds, but it is occasionally reported, such as in 2009, with a low frequency in a few European countries.

In Denmark, only a few outbreaks have been reported among pig herds within the last decade; 1999–2000 and 2012–2013 (Baggesen et al., 2000; Pedersen et al., 2015), and in both, it was impossible to identify the route of transmission and source of infection. It has been speculated that imported feed could have been the likely source of transmission or poorly disinfected livestock transportation vehicles or trailers returning to Denmark from low prevalence *S.* Choleraesuis endemic areas in Europe. Due to the methodologies applied of inadequate resolution and absence of European isolates, the linkage, however, was never confirmed. *S.* Choleraesuis has also been isolated from wild boars and it has been speculated that they could constitute a reservoir for transmission.

In this study, we tried to understand and elucidate the European transmission routes and the epidemiology of *S*. Choleraesuis var. Kunzendorf in the porcine environment including the contribution from wild boars. Available European strains from the last two decades were selected for whole genome sequencing and combined with a bioinformatics analysis that focused on the overall phylogeny, as well as the clonality within Danish herds and between the different organs of the same animal.

## Materials and Methods

### Bacterial Isolates

The study was conducted with the European Union Reference Laboratory network for antimicrobial resistance and the national reference laboratories within the area of antimicrobial resistance. An invitation requesting isolates from 2000 to 2013 was sent by email to a total of 118 contacts from the following countries: Iceland, France, Finland, Poland, Norway, Hungary, Spain, Luxemburg, Slovenia, Denmark, Austria, Croatia, Belgium, Cyprus, Czechia, Estonia, Germany, Greece, Iceland, Italy, Latvia, Lithuania, Netherlands, Slovakia, the United Kingdom, Portugal, Malta, Bulgaria, Romania, Switzerland, Serbia, and Turkey. The invitation requested isolates collected between 2000 and 2013. *S*. Choleraesuis isolates for the last two decades from the countries responding as having available isolates were included in the study. Additionally, the US CDC, USDA, and US FDA as well as to the Institute Pasteur in Saint Petersburg, Russia were invited into the study to supply strains outside of Europe to serve as out-group comparators. The swine isolates from the US FDA were selected based on PFGE profile over the same time period.

A total of 102 *S*. Choleraesuis isolates were collected from 12 countries including Austria (*n* = 5), Czechia (*n* = 4), Denmark (*n* = 44), Estonia (*n* = 12), France (*n* = 2), Germany (*n* = 7), Hungary (*n* = 3), Ireland (*n* = 2), Italy (*n* = 6), Lithuania (*n* = 5), Poland (*n* = 5), and the United States (*n* = 7). Most of the isolates available were included the study with a few exception (e.g., Poland, Germany, and the United States). The Danish strains originated from two large outbreaks in 1999–2000 and 2012–2013, and included strains isolated from the same herd and from different organs within the same animal to assess variability in the herds and within the animals.

All of the 102 *S*. Choleraesuis var. Kunzendorf (I 6,7:c:1,5) strains were identified and serotyped in the country of origin by conventional microbiological methodologies. The isolates originated mainly from pigs except for 12 isolates that were from wild boars and isolated from 1995 to 2013. The details of the isolates used in this study are shown in the Supplementary Table S1.

All pig tissue samples originated from already-existing diagnostic sample collected from the collaborating institutes. Permits were not necessary for obtaining wild boar tissues due to these samples being diagnosed for detection of *Trichinella spiralis* in relation to game killed for consumption. Ethical approval was not required for this study according to the local and national and guidelines.

### Whole Genome Sequencing

Genomic DNA was extracted from 102 pig and wild boar isolates using an Invitrogen Easy-DNA Kit^TM^ (Invitrogen, Carlsbad, CA, United States) and DNA concentrations were determined using the Qubit dsDNA BR assay kit (Invitrogen). The genomic DNA was prepared for Illumina pair-end sequencing using the Illumina (Illumina, Inc., San Diego, CA, United States) Nextera XT^®^ Guide 150319425031942 following the protocol revision C^1^. A sample of the pooled Nextera XT Libraries was loaded onto an Illumina HiSeq reagent cartridge using HiSeq Reagent Kit v2. The libraries were sequenced using an Illumina HiSeq platform.

Raw sequence data have been submitted to the European Nucleotide Archive^2^ under study accession no.: PRJEB18803. The raw reads were *de novo* assembled using the assembly pipeline (version 1.0) available from the Center for Genomic Epidemiology (CGE)^3^, which is based on the Velvet algorithm for *de novo* short reads assembly (Zerbino and Birney, 2008). A complete list of genomic sequence data is available in the Supplementary Table S1.

### Multilocus Sequence Typing, Antimicrobial Resistance Genes, and Plasmid Replicons

The assembled sequences were analyzed using *in silico* bioinformatics tools to confirm the serotype, identify the MLST (Multilocus sequence typing) sequence type (ST) for *Salmonella enterica*, plasmid replicons, and acquired antimicrobial resistance genes using the pipelines; Seqsero (version 1.1) (Zhang et al., 2015), MLST (version 1.7) (Larsen et al., 2012), PlasmidFinder (version 1.2) (Carattoli et al., 2014), and ResFinder (version 2.1) (Zankari et al., 2012) available from CGE^4^.

### Identification of Single Nucleotide Polymorphisms

Single nucleotide polymorphism (SNPs) were identified using the pipeline CSI phylogeny 1.4 (Leekitcharoenphon et al., 2012a; Kaas et al., 2014) available from CGE. The paired-end reads were mapped to the reference genome, *S*. Choleraesuis str. SC-B67 (AE017220, genome length 4,755,700 bp) (Chiu et al., 2005), using Burrows-Wheeler Aligner (BWA) version 0.7.2 (Li and Durbin, 2009). The SNPs were called using ‘mpileup’ module in SAMTools version 0.1.18 (Li et al., 2009). Subsequently, the SNPs were selected when they met the following criteria: (1) a minimum distance of 15 bps between each SNP (pruning), (2) a minimum of 10% of the average depth, (3) the mapping quality was above 30, (4) the SNP quality was more than 20, and (5) all indels were excluded. The qualified SNPs from each genome were concatenated to a single alignment corresponding to the position of the reference genome. The concatenated sequences were subjected to parsimony tree construction using PhyML (Guindon et al., 2010) with HKY85 substitution model and 100 bootstrap replicates.

### Temporal Bayesian Phylogenetic Structure

SNP alignments had been detected for significant recombination sites prior to reconstruction of the phylogenetic tree. We used a hidden Markov model tool called RecHMM (Zhou et al., 2014) to detect the clusters of sequence diversity that mark the recombination events within branches.

The Bayesian temporal phylogenetic reconstruction was completed based on the SNP alignment using BEAST (Bayesian evolutionary analysis sampling trees) version 1.8.4 (Drummond and Rambaut, 2007; Drummond et al., 2012; Suchard et al., 2018). Tested models with combinations of population size change and molecular clock were evaluated to determine the best-fit model. The final Bayesian temporal tree was constructed using a Bayesian skyline model as tree prior (Lewiński et al., 2013) and an uncorrelated relaxed lognormal clock (Drummond et al., 2006). Mutation rate and divergence time were estimated by BEAST.

The BEAST Markov chain Monte Carlo (MCMC) chains were simulated for 2,000 million steps and subsampled every 10,000 steps. The final single maximum clade credibility (MCC) was examined using TreeAnnotator (Drummond et al., 2012) with 10% of the MCMC steps discarded as burn-in. Statistical confidence was represented by the 95% highest posterior density (HPD) interval.

The transmission routes of *S*. Choleraesuis strains were carried out by the discrete phylogeographic analysis using a standard continuous-time Markov chain (CTMC) (Lemey et al., 2009) which is implemented in BEAST. A location-annotated MCC tree (MCC tree with DISCRETE traits) was imported to an interactive visualization tool called SpreadD3 (Bielejec et al., 2016) to visualize the transmission routes.

### Core Genes and Accessory Genes

Open reading frames (ORFs) were predicted based on the assembled genomes of *S*. Choleraesuis using Prodigal (Hyatt et al., 2010). Predicted genes were translated into amino acid sequences and aligned all-against-all by BLASTP (Altschul et al., 1990). Genes were grouped into the same gene family if the alignment length was at least 50% and percent similarity was at least 50% (the “50/50 rule”) (Tettelin et al., 2005). Gene families from all genomes were compared. Core genes were built from the intersection of gene families shared by every genome in the analysis (Tettelin et al., 2005; Leekitcharoenphon et al., 2012b). Any gene families that were not part of the core genes were considered as accessory genes.

Clusters of accessory genes were selected and aligned against the scaffold of plasmids associated with replicons previously found among the *S*. Choleraesuis strains and those of *S*. Choleraesuis SC-B67. This included the aligned plasmid scaffolds of *S*. Typhi R27 plasmid (AF250878) (Sherburne et al., 2000), *S*. Typhimurium plasmid R64 (AP005147) (Komano et al., 1987), *Serratia marcescens* plasmid R478 (BX664015) (Gilmour et al., 2004), *S*. Paratyphi C RKS4594 plasmid pSPCV (CP000858) (Liu et al., 2009), *S*. Typhimurium plasmid pSLT-BT (FN432031) (Kingsley et al., 2009), *S*. Typhimurium SL1344 plasmid pRSF1010_SL1344 (HE654726) (Kröger et al., 2012), *S*. Choleraesuis SC-B67 plasmid pSCV50 (NC_006855) (Yu et al., 2006) and *S*. Choleraesuis SC-B67 plasmid pSC138 (NC_006856) (Chiu et al., 2005).

### *Salmonella* Pathogenicity Islands

Sequences of the *Salmonella* pathogenicity islands (SPIs) were retrieved from Pathogenicity Island Database, PAI DB^5^. The sequences were aligned against all *S*. Choleraesuis genomes under study using BLASTN. The degree of similarity was displayed in a gradient heatmap.

## Results

### Multilocus Sequence Typing

The majority of *S*. Choleraesuis isolates (*n* = 81) were identified as ST-145 (formerly ST-1804) with the following loci and allele profile: *aroc*-36, *dnan*-31, *hemd*-35, *hisd*-14, *pure*-26, *suca*-6, *thra*-8. Twelve isolates from Estonia and one strain from the United States were single locus variants (*aroc*) of ST-145 identified by the following loci and alleles changes: *aroc*-129; ST-363 (formerly ST-1857) and *aroc*-34; ST-66 (formerly ST-1860). In addition, the remaining eight isolates from Germany, Italy and Austria were similarly single locus variant (*suca*) of ST-145 identified as ST-68 (formerly ST-1858) with the following loci and allele change; *suca*-34, (Supplementary Table S1). The eight isolates were excluded from the rest of the analysis due to the distant clonal relatedness.

### Antimicrobial Resistance Genes

**Figure 1:**
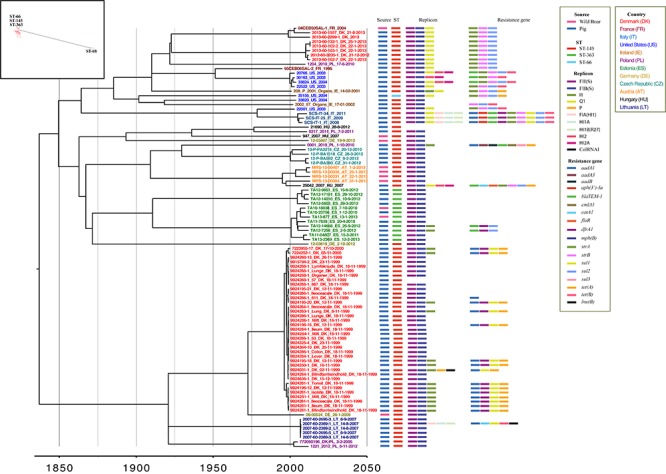
Bayesian maximum clade credibility (MCC) tree from BEAST for the 94 *S*. Choleraesuis isolates. The tree in the box is maximum likelihood tree of 102 *S*. Choleraesuis. The Bayesian-based temporal phylogenetic tree shows the most recent common ancestor of *S*. Choleraesuis in ∼1837 (95% credible interval, 1733–1983). Isolates are labeled by isolate identification number, country of origin, and date (day-month-year). The nodes are colored according to the country of the isolation.

The detection of antimicrobial resistance genes revealed that 58 isolates including 11 out of 12 strains from wild boars were pan-susceptible with no resistance genes identified among these isolates. One isolate from Denmark (9924286-1_611_DK_18_11_1999) contained a single resistance gene, *aad*A1, whereas the remaining 43 isolates contained multiple resistance genes (Figure 1 and Supplementary Table S1). The following antimicrobial resistance genes were identified among the isolates: *bla*_TEM-1_; ampicillin (AMP), *cat*A1, *cml*A1; chloramphenicol (CHL), *flo*R; florfenicol (FFN), *mph*(B) (2′-phosphotransferas II); erythromycin (ERY), *strA*, *str*B, *aad*A1, *aad*A3, *aad*B; streptomycin (STR), *sul*1, *sul*2, *sul*3; sulfamethoxazole (SMX), *tet*(A), *tet*(B); tetracycline (TET), *dfr*A1; trimethoprim (TMP), *aph(3′)-Ia*; gentamicin (GEN), and *lnu(*B*)*; lincomycin (LINCO) (Figure 1 and Supplementary Table S1). Interestingly, a few resistance gene patterns were observed among the isolates, such as among Danish outbreak strains from the 1999 to 2000 and 2012 to 2013. Two French isolates (04CEB50SAL-1_FR_2004 and 95CEB06SAL-2_FR_1995) and one Polish isolate (1204_2010_PL_17-6-2010) shared the same profile as strains of the latter outbreak (Figure 1).

### Plasmid Replicons

Almost all the isolates carried the plasmid replicons *inc*FII(S) and *inc*FIB(S) with the exception of three isolates from Hungary (21690_HU_28-8-2012), Italy (SCS-IT-21_IT_2009), and Denmark (9924288-1_57_DK_18-11-1999), which seemed to be plasmid-free.

A total of 11 different plasmid replicons in eight plasmids were detected among the isolates: *inc*FII(S), [*inc*FIB(S), *inc*FIA(HI1)], *inc*I1, *inc*Q1, *inc*P, [*inc*HI1A, *inc*HI1B(R27)], (*inc*HI2, *inc*HI2A), and *inc*ColRNAI (Figure 1 and Supplementary Table S1). The determination of resistance genes and plasmid replicons indicated that some resistance gene profiles and plasmid replicons among the isolates were linked. This included isolates of the Danish outbreaks, 1999–2000: *aad*A1, *dfr*A1, *sul*1, and *tet*(A) with *inc*I1 and 2012–2013 along with the French isolates: *str*A, *str*B, and *sul*2 with *inc*Q1 (Figure 1).

### Phylogenetic Tree and Temporal Phylogenetic Reconstruction Using BEAST

A maximum parsimony tree was constructed based on 4,254 SNPs identified from the 102 isolates aligned to the reference genome (Figure 1, in the box). The maximum parsimony tree consisted of distantly separated clusters according to MLSTs; separating ST-145, ST-363, and ST-66 from ST-68 with 1,847 SNPs. Due to the difference in clustering, a subset of 94 isolates was selected from the complex cluster of ST-145, ST-363, and ST-66 for further analysis.

The 94 isolates yielded a total of 2,342 SNPs that were subjected to temporal phylogenetic reconstruction using BEAST (Figure 1). The Bayesian MCC tree provided an estimated mutation rate of 2.14 × 10^-7^ SNPs/site/year, which is equivalent to 1.02 SNPs/genome/year. The most recent common ancestor (MRCA) was estimated to have emerged in 1837 (95% credible interval, 1733–1983).

The isolates were divided into two complex clusters with an average difference of 267 SNPs and resided in sub-clusters according to countries and neighbor countries of isolation (Figure 1). The exceptions were isolates from Germany, Poland, and Ireland that sporadically resided throughout the phylogenetic tree. It is noteworthy that the two historical Danish outbreaks clustered independently of each other in each of the main clusters. The four wild boar isolates from Austria, all isolated within a short time period clustered together, whereas the wild boar isolates from Estonia were clustered among Estonian pig isolates. Interestingly, both the wild boar and pig isolates from Estonia belonged to the same minor MLST, ST-363, but were quite diverse with an average of 62 SNP difference (Figure 1). The German and Hungarian wild boar isolates were more randomly dispersed throughout the phylogenetic tree and did not cluster to the pig isolates of the same countries.

The outbreak isolates from Denmark were distantly divided into different clade according to outbreak period and separated by 277 SNPs. The isolates of the earliest outbreak (1999–2000) had an average of 1 SNP difference (1–7 SNPs), in contrast to an average of 41 SNP difference (1–84 SNPs) for the latter outbreak (2012–2013). Two Danish isolates of the latter outbreak, 2013-60-1557_DK_21-8-2013 (Farm C) and 2013-60-2299-1_DK_2013 (Farm D) clustered with a French wild boar isolate from 2004 and were separated by 0 and 14 SNPs respectively. These strains also shared the same antimicrobial resistance genes and plasmid replicons.

Four groups of the isolates (9924281-1s, 9924284-1s, 9924286-1s, and 9924288-1s) from the earlier Danish outbreak were isolated from different organs of the same animal. Zero SNP difference was observed among the strains from different organs originating from the same pig, except for the isolates 9924281-1s, where an average of 1 SNPs (1–4 SNPs) was observed.

### Phylogeographic Analysis

**Figure 2:**
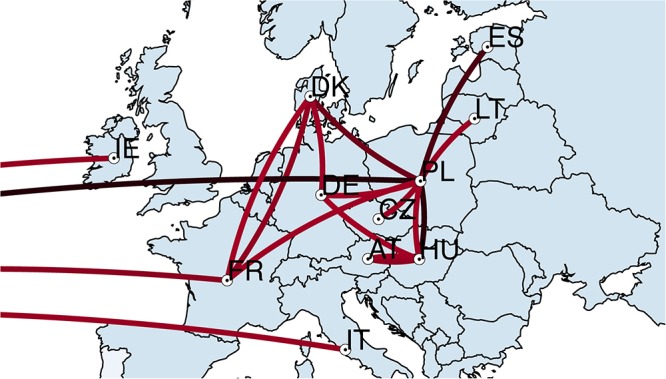
Transmission of *S*. Choleraesuis between countries. Discrete phylogeographic analysis of 94 *S*. Choleraesuis from 12 countries. The locations and transmission lines were obtained from the nodes and branches in a location-annotated MCC tree. The transmission lines on the left corner are the transmission lines associated with the United States. Black lines represent early transmission. Red lines represent recent transmission. IE, Ireland; FR, France; IT, Italy; DK, Denmark; DE, Germany; CZ, Czechia; AT, Austria; HU, Hungary; PL, Poland; LT, Lithuania; ES, Estonia.

All 94 *S*. Choleraesuis isolates from 12 countries were further analyzed in discrete phylogeographic analysis using a standard CTMC. The transmission of *S*. Choleraesuis between countries was summarized and illustrated in Figure 2. The earliest predicted dissemination (black lines) was between Poland and the United States followed by transmission between Poland and several countries; Hungary, Estonia, Denmark, Germany, Lithuania, and Czechia. More recent dissemination events (red lines) occurred between Denmark <=> France, Denmark <=> Germany, France <=> Poland, Hungary <=> Germany and Hungary <=> Austria. In addition, there were transmission events between the United States <=> Ireland, the United States <=> France and the United States <=> Italy. According to Food and Agriculture Organization of the United Nations (FAO) data^6^ of live animal trading from 1986 to 2016, we found that all the predicted transmission routes from this study (Figure 2) are concordant to the import/export network of pigs from FAO data (Supplementary Figure S1).

**Figure 3:**
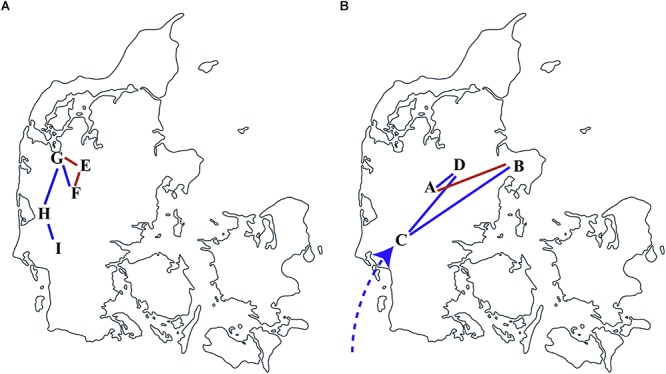
Transmission of *S*. Choleraesuis between farms in Denmark. Discrete phylogeographic analysis of *S*. Choleraesuis during 1999 **(A)** and 2013 **(B)** among farms in Denmark. The locations and transmission lines were obtained from the nodes and branches in a location-annotated MCC tree. The predicted transmission lines in red are concordant with the confirmed contact or trade between farms. The dashed line represents multiple introductions.

To illustrate the local transmission between farms of Danish *S*. Choleraesuis isolates, all Danish isolates together with geographical farm information were subjected to the discrete phylogeographic analysis using the CTMC. The spatial and temporal transmission of *S*. Choleraesuis isolates between nine different farms in Denmark was illustrated in Figure 3. The transmissions within the earliest outbreaks started in 1991 among farms E, F, G, H, and I (Figure 3A). The most recent dissemination occurred between farms B and A, farm B and C, farm A and D and farm C and D in 2012–2013 (Figure 3B). We have confirmed farm contacts between farms A and B, G and E and confirmed trade contact between farms F and E (Baggesen et al., 2000; Pedersen et al., 2015). These contacts supported our discrete phylogeographic analysis (Figure 3, red lines).

### Accessory Genes

**Figure 4:**
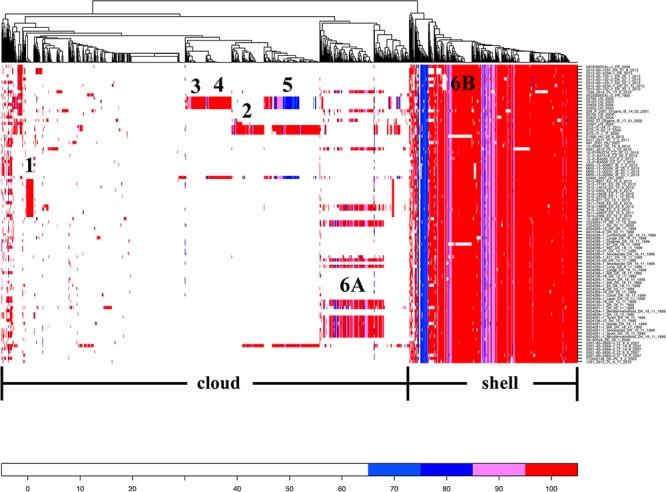
Heat map showing percent similarity of accessory genes across *S*. Choleraesuis isolates. Accessory genes are divided into genes that frequently found (shell) and genes that rarely found (cloud). There are six groups of accessory genes uniquely found in some isolates. The cut-off for being absent was 65% similarity. The isolates on the right were placed in the same order as the isolates in temporal phylogenetic tree in Figure 1.

From comparative genomics analysis, we found 4,211 core genes and 1,300 accessory genes among the 94 *S*. Choleraesuis isolates. The analysis of presence/absence of the accessory genes across the 94 isolates showed that the genes could be divided into 377 shell (genes that frequently found) and 923 cloud genes (genes that rarely found) (Figure 4). There were six groups of unique accessory genes (Figure 4 and Supplementary Table S2). The first group consisting of 17 genes was found only in strains from Estonia. The genes seemed not to be related to plasmids but suggested to be linked with ST-363 represented only by Estonian strains.

The second group consisted of 88 genes. These genes were present predominantly in Italian strains and an isolate from Lithuania (2007-60-2369-1_LT_14_8_2007). Forty-eight of the genes were similar to genes found in the plasmid scaffold of *Salmonella* Typhi R27. By mapping contigs from the Italian strains against the plasmid scaffold of R27, we found that the resistance genes *aad*A1, *aph*(3′)-*Ia, bla*_TEM-1_, *dfr*A1, *str*A, *sul*1, and *tet*(A) and replicons *inc*Q1, *inc*HI1, *inc*HI1A, and *inc*HI1B were located in the same contigs which was identified as R27 plasmid. In addition, 17 genes matched the plasmid scaffolds of *S.* Typhimurium R64, *Salmonella* Choleraesuis str. SC-B67 pSC138, *S.* Typhimurium pSLT-BT and *Serratia marcescens* R478.

The third group was found only in the isolates originating from the United States and contained 34 genes of which only one could be related to a plasmid, *Serratia marcescens* R478.

The fourth group, comprised of 62 genes, was found predominantly among the United States isolates and a Hungarian strain (25042_2007_HU_2007). The United States isolates all harbored the replicons *inc*Q1, *inc*HI2 and *inc*HI2A, of which the two latter replicons were originally from plasmid R478. Fifty-four of the accessory genes matched to exactly the plasmid scaffold of *Serratia marcescens* R478.

The fifth group contained 53 accessory genes, which all but one was similar to those found in the *S*. Typhi R27 plasmid. These genes were present mainly in isolates from the United States, Italy and an isolate from Hungary (25042_2007_HU_2007) and Lithuania (2007-60-2369-1_LT_14_8_2007).

The accessory genes from group 6 were subdivided into two groups (A and B) and found predominantly in some but solely Danish isolates from the earliest outbreak. The group 6A consisted of 134 genes of which 52, 38, 10, 5, and 1 genes were similar to the plasmid scaffold of *S.* Typhimurium R64, *S*. Choleraesuis str. SC-B67 pSC138, plasmids R478, R27 and *S*. Typhimurium plasmid pSLT-BT respectively. The seven genes from group 6B were non-related plasmid genes and they were almost in all isolates except for the most recent Danish outbreak isolates.

### *Salmonella* Pathogenicity Islands

**Figure 5:**
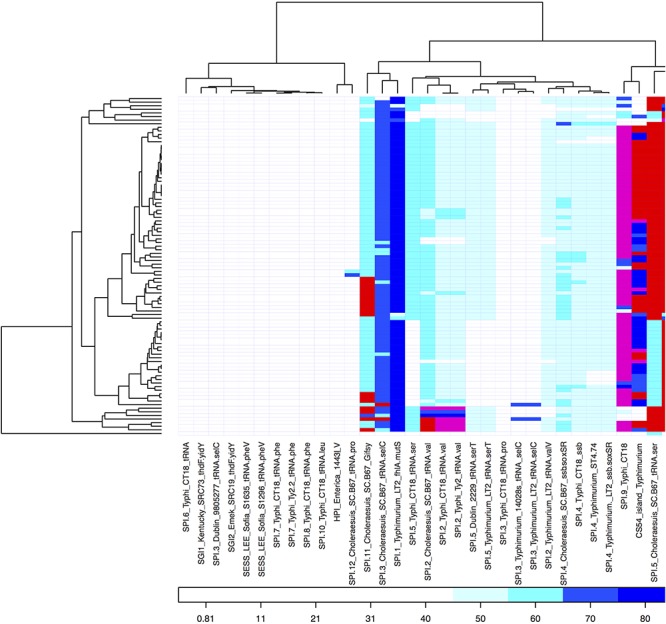
Heat map showing percent similarity of SPIs across *S*. Choleraesuis isolates. The cut-off for being absent was 45% similarity.

Forty sequences of SPIs were aligned against all the *S*. Choleraesuis isolates. The presence/absence of SPIs was shown in Figure 5. SPI-2, SPI-3, SPI-4, and SPI-11 were present with low percent identity (less than 65% identity) in all isolates. Five SPIs including the CS54 island were found in *S*. Choleraesuis isolates with high percent identity (greater than 95% identity). The five SPIs included SPI-1 present in *S*. Choleraesuis SC-B67, *S*. Typhi CT18, and *S*. Typhi Ty2; SPI-5 in *S*. Choleraesuis SC-B67; SPI-9 in *S.* Typhi CT18; SPI-13 in *S*. Gallinarum SGG-1, SGD-3, SGA-10; and SPI-14 from *S*. Gallinarum SGA-8, SGC-8.

## Discussion

In Denmark, outbreaks caused by *S.* Choleraesuis among pigs are rare with only two known outbreaks observed within the last two decades (Baggesen et al., 2000; Pedersen et al., 2015). The two outbreaks have previously been described based on a much smaller data set than in this study, and that description was inconclusive in terms of the origin of the infection (Baggesen et al., 2000; Pedersen et al., 2015). In this study, the number of isolates was expanded to include more isolates from the two Danish outbreaks as well as from other European countries.

The results of the spatial analysis of this study were concordant with the national trade- associated routes of transmission that were previously proposed between farms in Denmark. Trade contact had also been confirmed epidemiologically between farms G–E and farms F–E.

Of interest, farm C had, prior to the outbreak, imported corn directly delivered by vehicles, without involving a feed company and heat treatment, from European countries such as Poland and Germany where *S*. Choleraesuis is endemic. Farm C only produced slaughter pigs and sold pigs for on-growing (Pedersen et al., 2015). We found that the isolate from farm C (2013-60-1557_DK_21-8-2013) did not cluster closely with the other isolates from the same outbreak. This isolate was genetically closer related to another wild boar isolate from France separated by zero SNP with 9 years apart. This suggests that an outbreak strain has been circulating in other countries for several years before it was transmitted to and responsible for the later outbreak in Denmark. The strain could at the time of the outbreak via an intermediate transmission be the source of the outbreak due to vehicles crossing the border to Denmark without properly being cleaned and disinfected as earlier suggested (Pedersen et al., 2015). If this is the case and the vehicle contamination is from serious pig disease with high mortality rate such as African swine fever, it would heavily affect the pig industry. It is important to properly disinfect livestock transportation vehicles to prevent contamination and transmission from vehicles.

The isolates from the two Danish outbreaks were different, separated by more than 277 SNPs, and did not harbor either the same resistance genes or plasmid replicons indicating that the strains from the initial outbreak did not persist in Denmark. The isolates of the earliest outbreak were more clonal with an average of a one-SNP difference indicating one introduction. Twenty-one of the isolates were isolated from different organs in the same pigs. In one animal, isolates 9924281-1s differed by 1 SNP on average (1–4 SNPs). This indicates that mutation can occur even though the clonal strains are from the same animal.

In contrast to the earlier outbreak (1999–2000), the isolates of the latter outbreak (2012–2013) were more diverse with 41 SNP difference indicating multiple introductions and extensive trade. In the latter outbreak, the pigs were introduced to multiple clones of *S*. Choleraesuis from complex samples, for instance imported contaminated feed (Pedersen et al., 2015).

The mutation rate of the *S*. Choleraesuis was 1.02 SNPs/genome/year. The rate was similar to previous studies in a series of egg-associated outbreaks of *S*. Typhimurium (1–2 SNPs/genome/year) (Hawkey et al., 2013) and other studies of global dissemination of *S*. Typhimurium (1–2 SNPs/genome/year) (Okoro et al., 2012; Leekitcharoenphon et al., 2016).

The temporal phylogenetic tree also showed that *S*. Choleraesuis isolates from different countries were clustered according to countries and neighboring countries of isolation, with the exception of the isolates from Germany, Poland and Ireland. This indicates degrees of cross-border transmission. Isolates from the United States resided in the same clusters with those from Ireland and Italy. Nonetheless, the United States isolates do not share the same pattern of resistance genes nor plasmid replicons. The isolates from the United States were intentionally used as an out-group, but the data suggest connections between the strains from the United States and the strains from Ireland, France, Italy and Poland. It could be explained by a possible closer connection between the countries, as Ireland is more geographically remote from other European countries. The phylogeographic analysis showed that the transmission of *S*. Choleraesuis between countries was concordant to the trading network of pigs.

Only three wild boar isolates from Austria clustered together. The other wild boar isolates clustered with isolates from pig, indicating a wildlife reservoir and a potential transmission between wild boar and pigs. Wild boar is an opportunistic and scavenger species, and *S*. Choleraesuis may become colonized in wild boar after contacting with pig farms and manure from pig farms. Since the isolates are pan-susceptible (except the wild boar isolate from France), colonization in wild boar populations may have occurred decades ago. For pigs, wild boars are not just any reservoir like crows or rodents, but the ancestor stock, capable of interbreeding and presumably having a similar gut flora. Other studies on pathogens transmission between wild boars and pigs also found that wild boars seem to play an important ecological role in the dissemination of pathogen between domestic pig and wild boar populations (Frantz et al., 2015; Jori et al., 2016).

Estonian strains formed a unique cluster indicated by a unique ST type (ST-363). This might be that the European type of *S*. Choleraesuis has not reached Estonia or vice versa. This should be confirmed by analyzing isolates from the western part of Russia to investigate what ST types are closer to Estonia and European strains.

The European and the United States isolates of this study are different from those of South East Asia. In South East Asia, many *S*. Choleraesuis isolates have been described as being resistant to extended spectrum cephalosporins (ESC) harboring either *bla*_CMY -2_, *bla*_CTX-M-14_ or *bla*_CTX-M-9_ genes located on various plasmids such as *inc*A/C or *inc*FIIA/*inc*FrepB plasmids. In addition to being ESC producers, some of these strains also harbor resistance genes to first line antimicrobials and similar to those in this study. This includes the chloramphenicol resistance acetyltransferase gene *cml*A, sulfonamide resistance gene *sul*3, aminoglycoside resistance gene *aad*A1 and the tetracycline resistance gene *tet*(B) (Sirichote et al., 2010).

The presence/absence of accessory genes across the isolates showed distant patterns found predominantly in some of the isolates. The accessory genes also support the clustering of *S*. Choleraesuis in the phylogenetic tree. Accessory genes are often associated with plasmids, and those in *S*. Choleraesuis were related to plasmids from *Salmonella enterica*, *Serratia marcescens*, or *S*. Typhi R27. These plasmids are known to harbor multiple resistance genes such as *aad*A1, *aph(3′)-Ia, bla*TEM-1, *dfr*A1, *str*A, *sul*1, *and tet*(A). Plasmids are also known to be linked to virulence as well as SPIs that linked to virulence in *Salmonella*.

SPIs are horizontally acquired genomic elements responsible for the pathogenesis of *Salmonella*. The five SPIs present in the *S*. Choleraesuis strains all contributed to the virulence. SPI-1 encodes a type III secretion system (T3SS) that is required for invasion to mediate the contact-dependent translocation of effector proteins into host cells (Galán, 2001), and SPI-5 encodes effector proteins for the T3SS. SPI-9 encodes putative virulence factors, a type I secretion system and a large RTX-like protein (Parkhill et al., 2001). A recent study showed that SPI-13 may involve in pathogenesis and the host adaptation/restriction of *Salmonella* serovars (Elder et al., 2016). The CS54 island carries genes, for example *shdA* gene, required for intestinal persistence in the host (Kingsley et al., 2003). These are all virulence factors enabling *S*. Choleraesuis the propensity to cause extraintestinal infections in humans and pigs.

This study was limited by the available small number of wild boar isolates and the sampling regime. Nevertheless, we believe the data are sound and the speculations and conclusions proposed are legitimate.

## Conclusion

The results suggest that the two Danish outbreaks are independent; the outbreak of 1999–2000 was from a single unknown introduction, whereas the outbreak of 2012–2013 was from multiple introductions. A French wild boar isolate from 2004 was the closest related strain to the outbreak of 2012–2013 indicating a possible persistent reservoir in France before 2004 or any other sources common to France and Denmark. In an outbreak situation, it is paramount to know from where the outbreak originated. Knowledge of an outbreak’s origin will allow the investigator to focus on a special geographical area, source or trade pattern rather than losing valuable time in a random search. The origin of isolates causing the outbreaks cannot be properly investigated or inferred without comparing genomic data from different geographic areas and different origins (e.g., humans, wildlife, farmed animals). Therefore it is vital to have WGS data from different sources or countries shared in real time.

The wildlife reservoir including transmission between wild boar and pig was identified as another potential route of *S*. Choleraesuis transmission. A low degree of mutation can be observed between clonal strains originating from different organs of the same animal. The *S*. Choleraesuis isolates from Europe and the United States are not ESC-producing isolates, but they harbor multiple resistance genes and plasmid replicons, which are associated with accessory genes. SPIs, plasmids and accessory genes are related to the virulence of *S*. Choleraesuis.

Cross-border and trade transmission via contaminated vehicles and feed seem to be reasonable explanation on how *S*. Choleraesuis was disseminated in Europe and the cause of the outbreak in Denmark. On the basis of these findings, we conclude that properly disinfected livestock vehicles and improved quality control of livestock feed could help to prevent contamination from and outbreaks of *S*. Choleraesuis.

## Author Contributions

PL, GS, FA, and RH critically revised the manuscript, conceived and designed the experiments. GS, CL, AB, IS, DW, RS, SZ, AB, CK, AK, JS, TČ, CS, and KP performed field sampling. PL analyzed the data. PL, GS, FA, RH, CL, and AB wrote the manuscript. All authors read and accepted the manuscript.

## Conflict of Interest Statement

The authors declare that the research was conducted in the absence of any commercial or financial relationships that could be construed as a potential conflict of interest.

## Footnotes

1 http://support.illumina.com/downloads/nextera_xt_sample_preparation_guide_15031942.html

2 http://www.ebi.ac.uk/ena

3 https://cge.cbs.dtu.dk/services/Assembler/

4 http://cge.cbs.dtu.dk/services/all.php

5 www.paidb.re.kr

6 http://www.fao.org/faostat/en/\#data/QA

## Supplementary Material

Figure S1 Trade network of pigs accumulated from 1986 to 2016. The trade data were selected according to the transmission route from this study. The source of the arrow represents exporter and the arrow points to importer. The trade network showed **(A)** number of pigs and **(B)** ton of pigs. The weight of arrow is proportional to number and tons of export/import pigs.

Table S1 Genomic information including ENA accession number, MLST type, resistance genes and plasmid replicons.

Table S2 Alignment details of the accessory genes from the six groups.
