# Supplementary material for: Cross-Border Transmission of Salmonella Choleraesuis var. Kunzendorf in European Pigs and Wild Boar: Infection, Genetics, and Evolution
Source: Front Microbiol. 2019 Feb 6;10:179. doi: 10.3389/fmicb.2019.00179 (PMC6373457; doi:10.3389/fmicb.2019.00179)
Supplement: Figure S1 — Trade network of pigs accumulated from 1986 to 2016. The trade data were selected according to the transmission route from this study. The source of the arrow represents exporter and the arrow points to importer. The trade network showed (A) number of pigs and (B) ton of pigs. The weight of arrow is proportional to number and tons of export/import pigs. [file Data_Sheet_1.PDF]

(A)

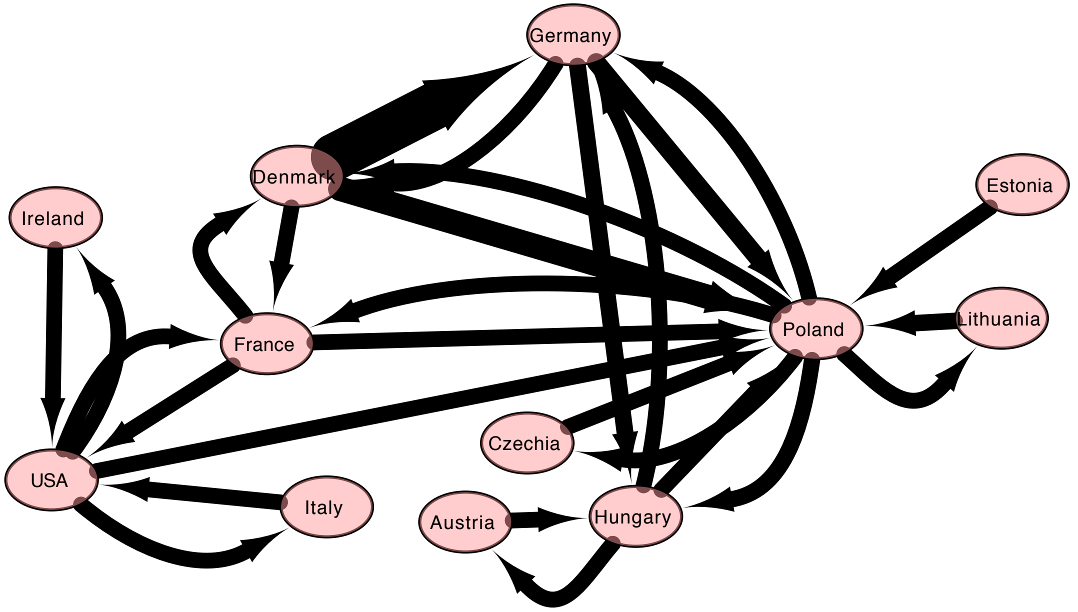

(B)

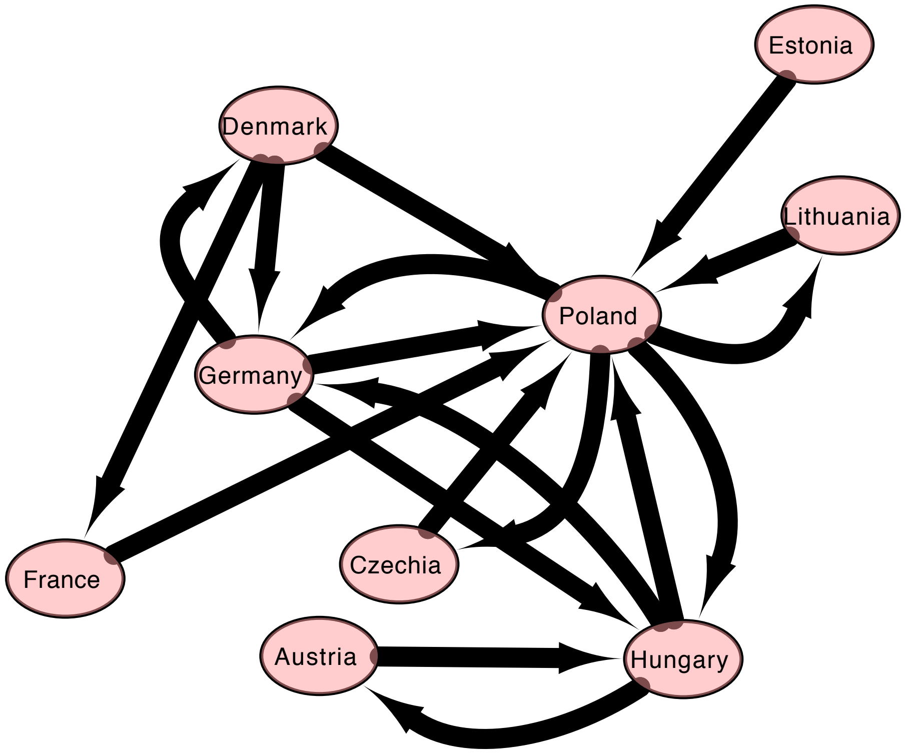

Supplementary figure 1. Trade network of pigs accumulated from 1986 - 2016. The trade data were selected according to the transmission route from this study. The source of the arrow represents exporter and the arrow points to importer. The trade network showed (A) number of pigs and (B) ton of pigs. The weight of arrow is proportional to number and tons of export/import pigs.
